# Supplementary material for: Synchrony between midbrain gene transcription and dopamine terminal regulation is modulated by chronic alcohol drinking
Source: Nat Commun. 2025 Feb 25;16:1944. doi: 10.1038/s41467-025-56715-y (PMC11850823; doi:10.1038/s41467-025-56715-y)
Supplement: Supplementary file 1 — Supplementary Information [file 41467_2025_56715_MOESM1_ESM.pdf]

Supplementary Materials for

Synchrony between midbrain gene transcription and dopamine terminal regulation is modulated by chronic alcohol drinking

Zahra Z. Farahbakhsh[1], Katherine M. Holleran[2], Jonathon P. Sens[2], Steve C. Fordahl[3], Madelyn I. Mauterer[2], Alberto J. López[1], Verginia C. Cuzon Carlson[4], Drew D. Kiraly[2], Kathleen A. Grant[4], Sara R. Jones[2], Cody A. Siciliano[1]✉

[1]Department of Pharmacology, Vanderbilt Brain Institute, Vanderbilt Center for Addiction Research, Vanderbilt University, Nashville, TN 37232, USA.
[2] Wake Forest University School of Medicine, Department of Physiology and Pharmacology, Winston-Salem, NC 27157, United States
[3] The University of North Carolina at Greensboro, The Department of Nutrition, Greensboro, NC 27412, United States
[4] Oregon National Primate Research Center, Oregon Health & Science University, Division of Neuroscience, Portland, Oregon, USA

Corresponding author:
Cody A Siciliano
✉ cody.siciliano@vanderbilt.edu

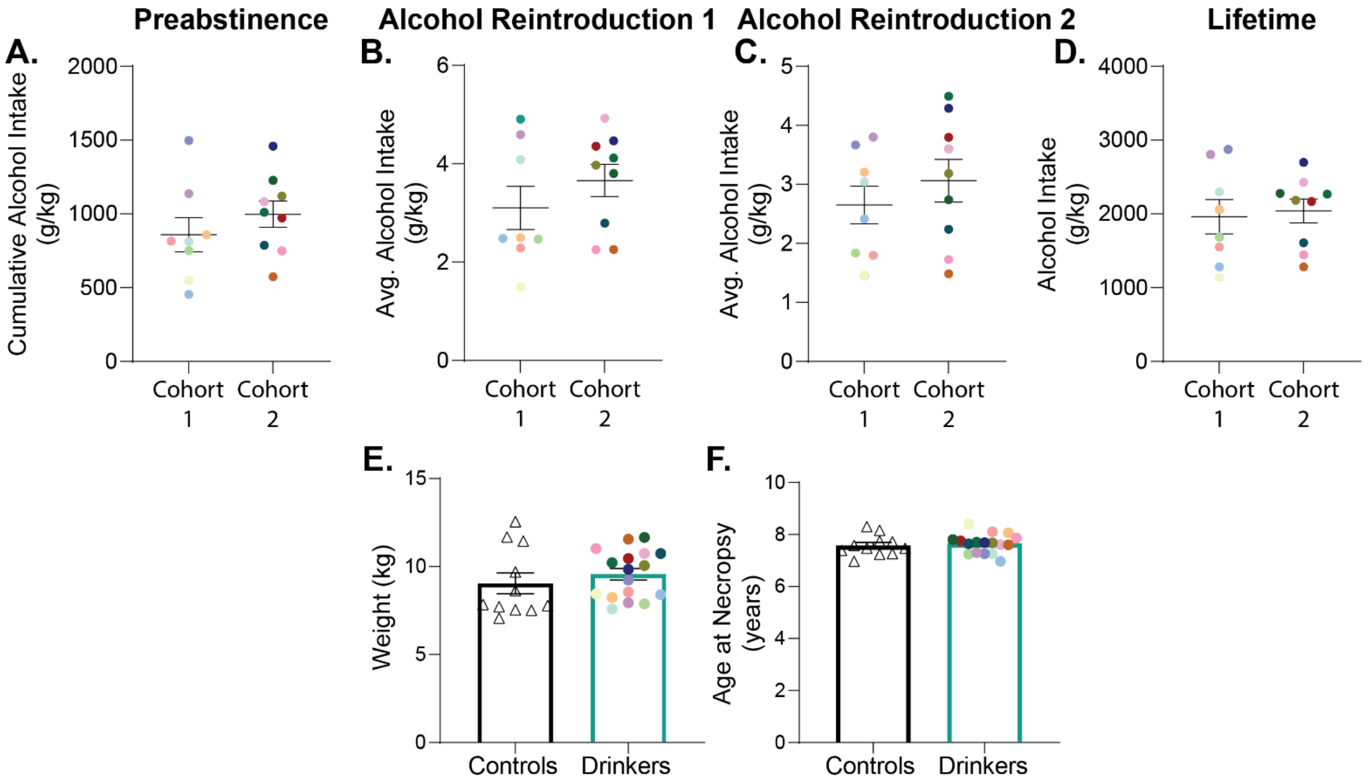

Supplemental Figure 1. No differences in alcohol consumption between cohorts. There was no difference between cohort 1 and cohort 2 in **(A)** cumulative alcohol intake during the first 12 months of open access (unpaired t-test; $t_{15} = 0.96$, $p = 0.35$), **(B)** the average daily alcohol consumption during the first alcohol reintroduction period (unpaired t-test; $t_{15} = 1.03$, $p = 0.32$), **(C)** the average daily alcohol consumption during the second alcohol reintroduction period (unpaired t-test; $t_{15} = 0.84$, $p = 0.41$), **(D)** or in the lifetime alcohol intake (unpaired t-test; $t_{15} = 0.28$, $p = 0.78$). **(E)** There was no difference in the weight of controls compared to drinkers at the time of necropsy (unpaired t-test; $t_{26} = 0.8255$, $p = 0.4166$). **(F)** There was no difference in age at necropsy between the controls and drinkers (unpaired t-test; $t_{26} = 0.4867$, $p = 0.6306$). (controls: n = 11; drinkers: n = 17; cohort 1: n = 8; cohort 2: n = 9) Values indicate mean ± SEM, with colors indicating individual subjects matched through the figure. All statistical tests were two-tailed.

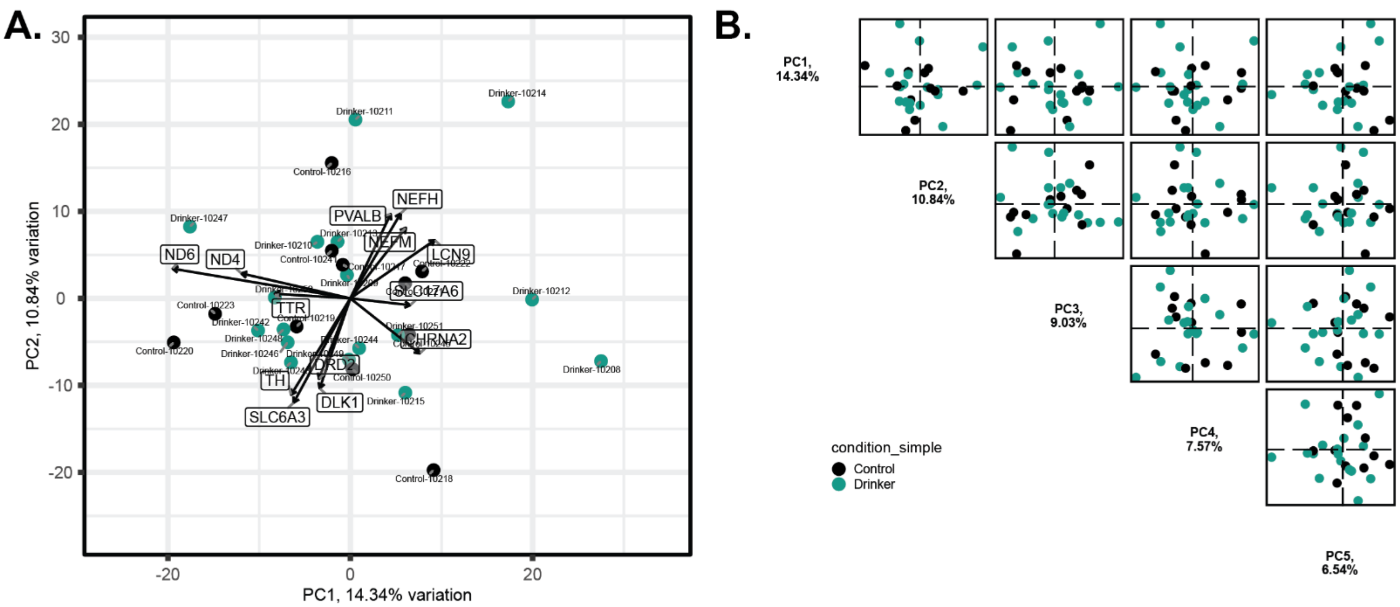

Supplemental Figure 2. Dimensionality reduction of gene expression via principal component analysis did not produce segregation between drinkers and controls. (A) There was no appreciable segregation between drinkers and controls across the first 2 principal components. The top genes that influenced the variance are presented along with a vector demonstrating the gene effect through length and the association with other genes through direction. The top genes include genes encoding the dopamine transporter (*SLC6A3*), the D2-type dopamine receptor (*DRD2*), and the rate-limiting enzyme of dopamine synthesis (*TH*). **(B)** Visualization of the top 5 principal components showed no appreciable segregation between groups. (controls: n = 11; drinkers: n = 17)

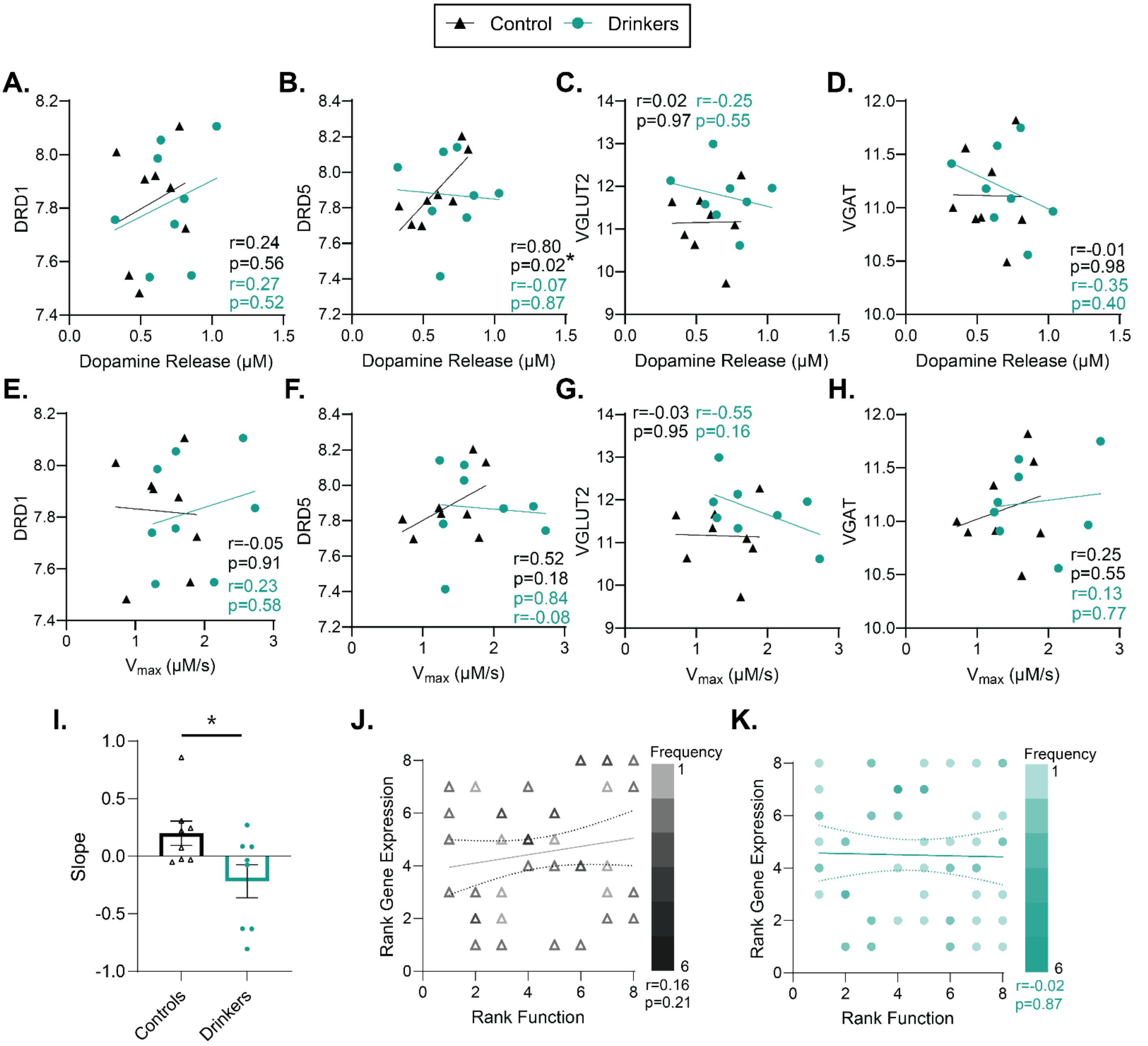

Supplemental Figure 3. Synchrony between upstream transcription with downstream dopamine terminal function was specific to homosynaptic regulators of the dopamine system. (A-H) Expression of genes encoding for receptors and transporters in the VTA which are enriched in non-dopaminergic cells was compared to terminal dopamine release and reuptake in the NAc of the same subjects. The best-fit linear regression is plotted for each group and Pearson's correlation coefficient r and associated p values are reported as an inset. **(A)** There was no correlation between dopamine receptor 1 (*DRD1*) gene expression and dopamine release in either group. **(B)** Dopamine receptor 5 (*DRD5*) gene expression was positively correlated with dopamine release in controls, but not drinkers. **(C)** Expression of vesicular glutamate transporter 2 (*VGLUT2*) did not correlate with dopamine release. **(D)** Vesicular GABA transporter (*VGAT*) did not correlate with dopamine release. **(E-H)** There were no correlations found between V_{max} and expression of *DRD1, DRD5, VGLUT2*, or *VGAT*. **(I)** The mean slope was greater in controls (unpaired t-test; t_{14} = 2.332, p = 0.0351), but neither group differed from 0 (one sample t-test; controls: t_7 = 1.884, p = 0.1016; drinkers: t_7 = 1.509, p = 0.1751). **(J-K)** Values are presented for each group with the color of the icon indicating the frequency of that

coordinate, and the best-fit linear regression is shown with a 95% confidence band and Spearman's correlation r and p values are indicated. **(J)** There is no correlation between upstream expression of non-dopamine neuron specific receptors and transporters and downstream terminal release dynamics in control subjects. **(K)** There is also no correlation between expression of these genes and accumbal dopamine dynamics after voluntary ethanol consumption and abstinence. Unless otherwise indicated, values indicate mean ± SEM. All statistical tests were two-tailed. (controls: n = 8; drinkers: n = 8) (* = $p \leq 0.05$, ** = $p \leq 0.01$, *** = $p \leq 0.001$, **** = $p \leq 0.0001$)

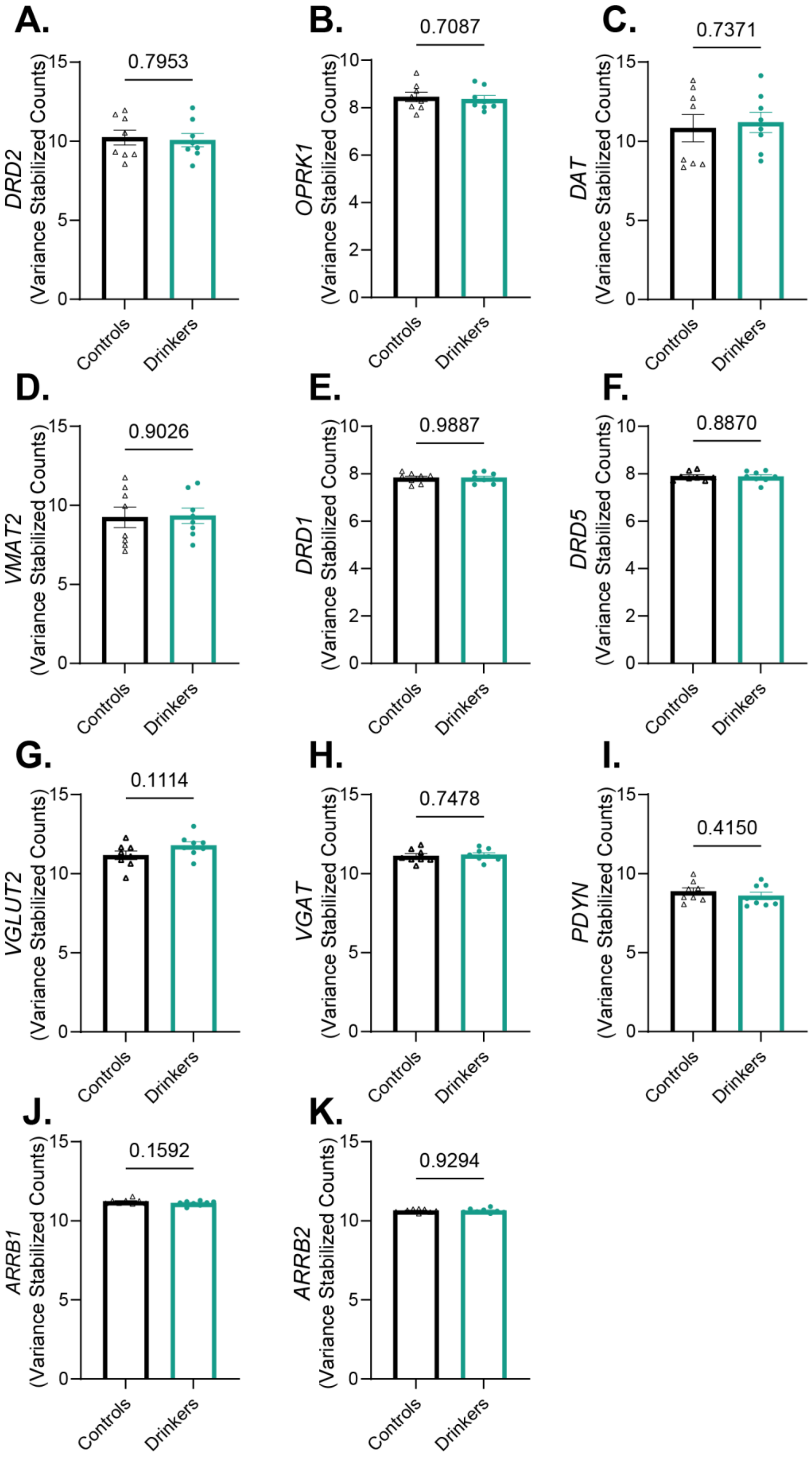
A.
DRD2
(Variance Stabilized Counts)
0.7953
B.
OPRK1
(Variance Stabilized Counts)
0.7087
C.
DAT
(Variance Stabilized Counts)
0.7371
D.
VMAT2
(Variance Stabilized Counts)
0.9026
E.
DRD1
(Variance Stabilized Counts)
0.9887
F.
DRD5
(Variance Stabilized Counts)
0.8870
G.
VGLUT2
(Variance Stabilized Counts)
0.1114
H.
VGAT
(Variance Stabilized Counts)
0.7478
I.
PDYN
(Variance Stabilized Counts)
0.4150
J.
ARRB1
(Variance Stabilized Counts)
0.1592
K.
ARRB2
(Variance Stabilized Counts)
0.9294
Controls
Drinkers

Supplemental Figure 4. Long-lasting functional changes and changes in transcriptional synchrony occur without significant differences in gene expression between groups. Drinkers and controls did not differ in VTA gene expression of **(A)** the dopamine receptor 2 (*DRD2*) (unpaired t-test; $t_{14} = 0.2644$, p = 0.7953), **(B)** the kappa opioid receptor (*OPRK1*) (unpaired t-test; $t_{14} = 0.3813$, p = 0.7087), **(C)** the dopamine transporter (*DAT*) (unpaired t-test; $t_{14} = 0.3425$, p = 0.7371), **(D)** the vesicular monoamine transporter 2 (*VMAT2*) (unpaired t-test; $t_{14} = 0.1246$, p = 0.9026), **(E)** the dopamine receptor 1 (*DRD1*) (unpaired t-test; $t_{14} = 0.01468$, p = 0.9887), **(F)** the dopamine receptor 5 (*DRD5*) (unpaired t-test; $t_{14} = 0.1447$, p = 0.8870), **(G)** the vesicular glutamate transporter 2 (*VGLUT2*) (unpaired t-test; $t_{14} = 1.699$, p = 0.1114), **(H)** the vesicular GABA transporter (*VGAT*) (unpaired t-test; t_{14} =0.3280, p = 0.7478), **(I)** prodynorphin (*PDYN*) (unpaired t-test; $t_{14} = 0.8400$, p = 0.4150), **(J)** β-arrestin 1 (*ARRB1)* (unpaired t-test; $t_{14} = 1.49$, p = 0.16), **(K)** or β-arrestin 2 *(ARRB2)* (unpaired t-test; $t_{14} = 0.09$, p = 0.93). (controls: n = 8; drinkers: n = 8) Values indicate mean ± SEM. All statistical tests were two-tailed.

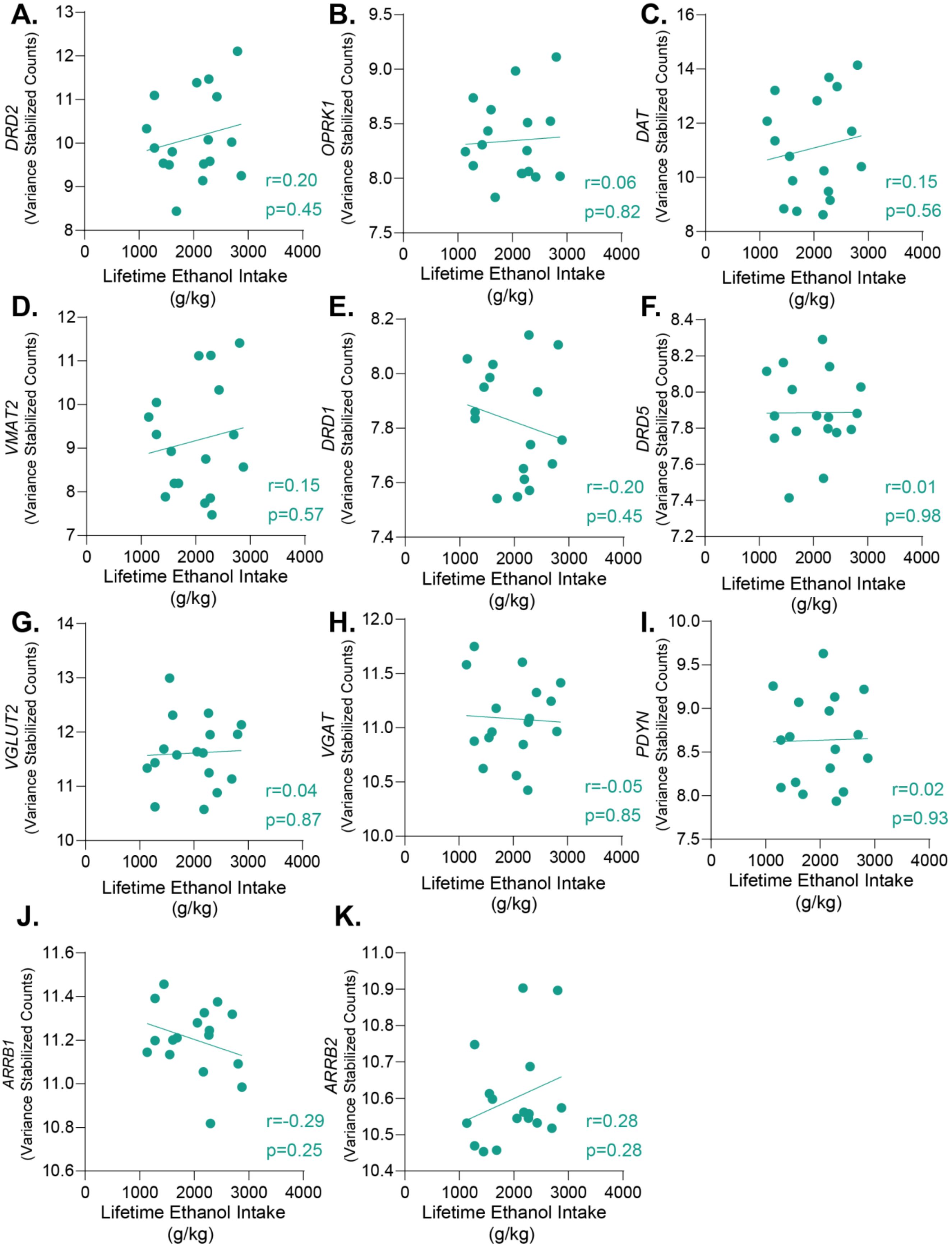
A.
DRD2
(Variance Stabilized Counts)
r=0.20
p=0.45
B.
OPRK1
(Variance Stabilized Counts)
r=0.06
p=0.82
C.
DAT
(Variance Stabilized Counts)
r=0.15
p=0.56
D.
VMAT2
(Variance Stabilized Counts)
r=0.15
p=0.57
E.
DRD1
(Variance Stabilized Counts)
r=-0.20
p=0.45
F.
DRD5
(Variance Stabilized Counts)
r=0.01
p=0.98
G.
VGLUT2
(Variance Stabilized Counts)
r=0.04
p=0.87
H.
VGAT
(Variance Stabilized Counts)
r=-0.05
p=0.85
I.
PDYN
(Variance Stabilized Counts)
r=0.02
p=0.93
J.
ARRB1
(Variance Stabilized Counts)
r=-0.29
p=0.25
K.
ARRB2
(Variance Stabilized Counts)
r=0.28
p=0.28
Lifetime Ethanol Intake
(g/kg)

Supplemental Figure 5. Expression of genes associated with functional changes did not correlate with lifetime ethanol intake. Lifetime ethanol intake of drinkers did not correlate with gene expression of **(A)** the dopamine receptor 2 (*DRD2*), **(B)** the kappa opioid receptor (*OPRK1*), **(C)** the dopamine transporter (*DAT*), **(D)** the vesicular monoamine transporter 2 (*VMAT2*), **(E)** the dopamine receptor 1 (*DRD1*), **(F)** the dopamine receptor 5 (*DRD5*), **(G)** the vesicular glutamate transporter 2 (*VGLUT2*), **(H)** the vesicular GABA transporter (*VGAT*), **(I)** or prodynorphin (*PDYN*), **(J)** β-arrestin 1 (*ARRB1)*, **(K)** or β-arrestin 2 *(ARRB2)*. The best-fit linear regression is plotted for each group and Pearson's correlation coefficient r and associated p values are reported as an inset. All statistical tests were two-tailed. (n = 17)

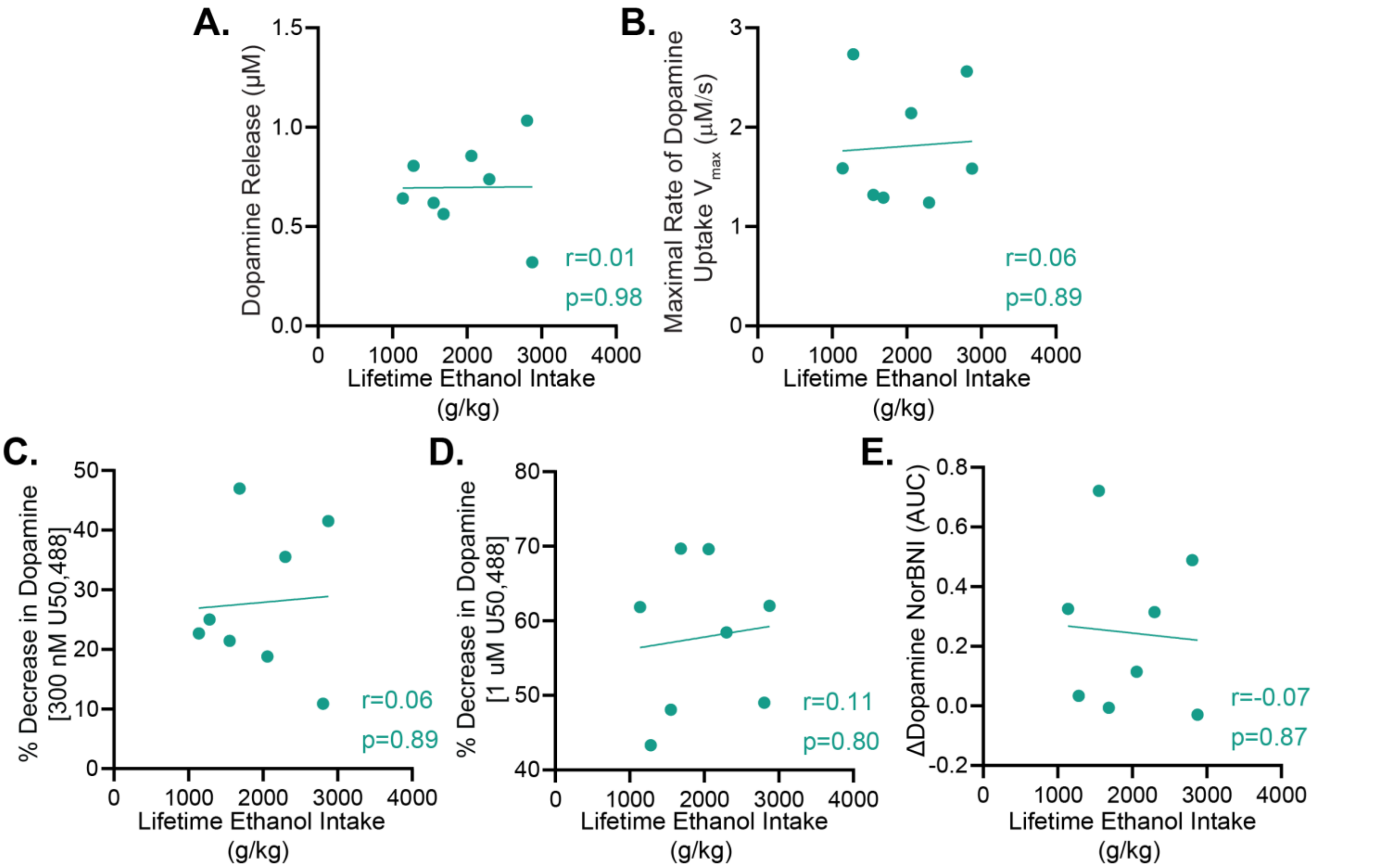

Supplemental Figure 6. Functional changes at the dopamine terminal did not correlate with lifetime ethanol intake. Lifetime ethanol intake did not correlate with **(A)** one pulse dopamine release, **(B)** the maximal rate of dopamine reuptake (V_{max}) **(C)** the percent decrease in dopamine release with bath application of 300 nM U50,488, **(D)** the percent decrease in dopamine release with bath application of 1 µM U50,488, **(E)** or the percent change in the area under the curve (AUC) of dopamine release with bath application of norbinaltorphimine (NorBNI). The best-fit linear regression is plotted for each group and Pearson's correlation coefficient r and associated p values are reported as an inset. All statistical tests were two-tailed. (n = 8)

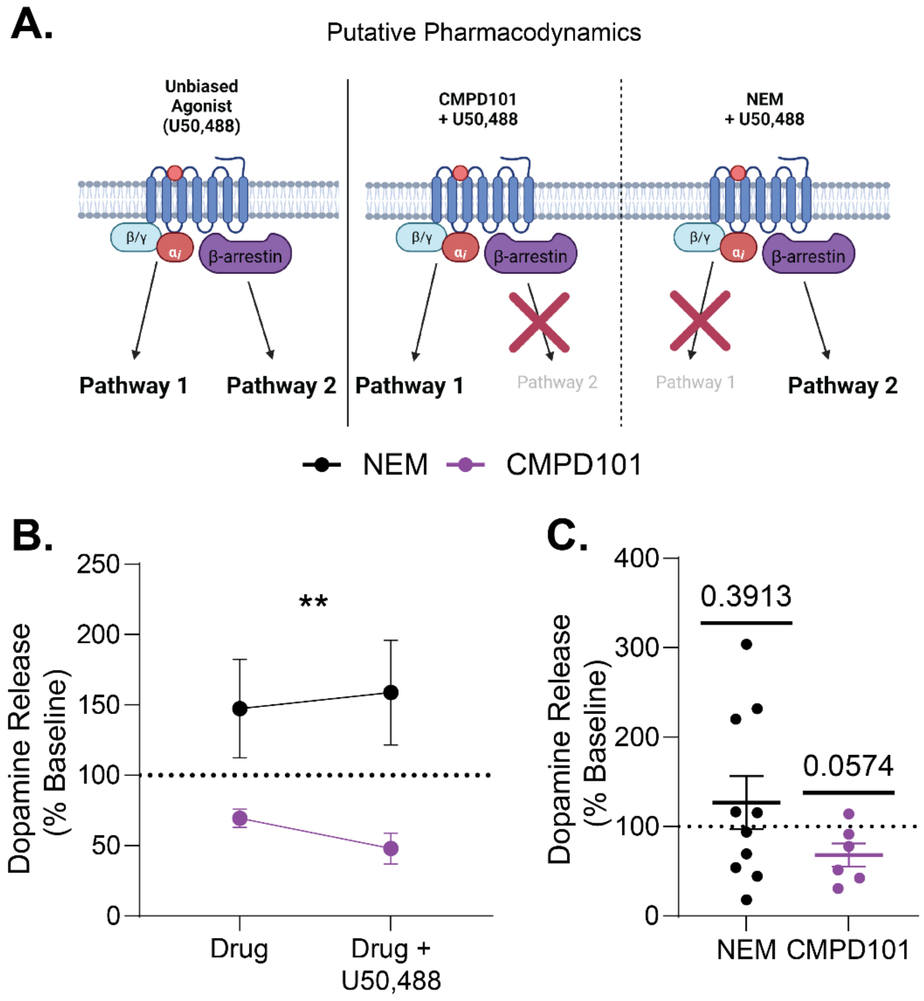

Supplemental Figure 7. KOR inhibition of terminal dopamine release may be G protein pathway mediated. (A) Experimental rationale for assessing whether KOR control over dopamine release is mediated by the G protein or β-arrestin pathway. An unbiased agonist, like U50,488, will activate both pathways similarly. Compound 101 (CMPD101) inhibits the β-arrestin pathway, thus, in its presence, application of an agonist would only activate the G protein pathway. N-ethylmaleimide (NEM), in contrast, inhibits G protein signaling, therefore in the presence of an agonist, only the β-arrestin pathway would be activated **(B)** CMPD101 or NEM and U50,488 (a KOR agonist) were applied consecutively and demonstrated differential effects on dopamine release normalized to pre-drug levels (two way ANOVA; wash-on: $F_{1, 26} = 0.02505$, $p = 0.8755$; drug: $F_{1, 26} = 8.645$, $p = 0.0068$; wash-on x drug: $F_{1, 26} = 0.2644$, $p = 0.6114$). **(C)** To account for their different effects, dopamine release after cumulative U50,488 bath application was then normalized to values seen with each of the drugs alone. Application of U50,488 had no effect when NEM was present (one sample t-test; $t_9 = 0.9005$, $p = 0.3913$), but had a trending decrease in dopamine release in the presence of CMPD101 (one sample t-test; $t_5 = 2.458$, $p = 0.0574$) suggesting that G protein activity is necessary to KOR-mediated inhibition of dopamine release. Values indicate mean ± SEM. All statistical tests were two-tailed. (* $p \leq 0.05$, ** $p \leq 0.01$, *** $p \leq 0.001$, **** $p \leq 0.0001$) (NEM: n = 10; CMPD101: n = 6) Created in BioRender. Lab, S. (2024) https://BioRender.com/a10l505

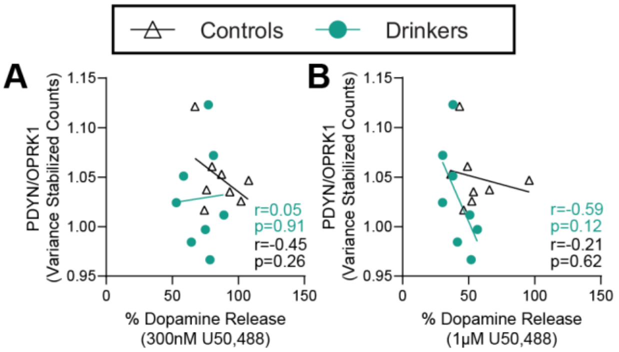

Supplemental Figure 8. NAc KOR sensitivity is not associated with the ratio of ligand to receptor gene expression in the VTA. The relationship between the potency (300 nM) and efficacy (1 µM) of U50,488 in inhibiting dopamine release in the NAc and ligand/receptor ratio of *PDYN* and *OPRK1* expression in the VTA. The best-fit linear regression is plotted for each group and Pearson's correlation coefficient r and associated p values are reported as an inset. **(A)** There was no relationship between *PDYN/OPRK1* expression and the amount of dopamine released in the presence of 300 nM U50,488. **(B)** There was no relationship between *PDYN/OPRK1* expression and the amount of dopamine released in the presence of 1 µM U50,488. All statistical tests were two-tailed. (controls: n = 8; drinkers: n = 8)

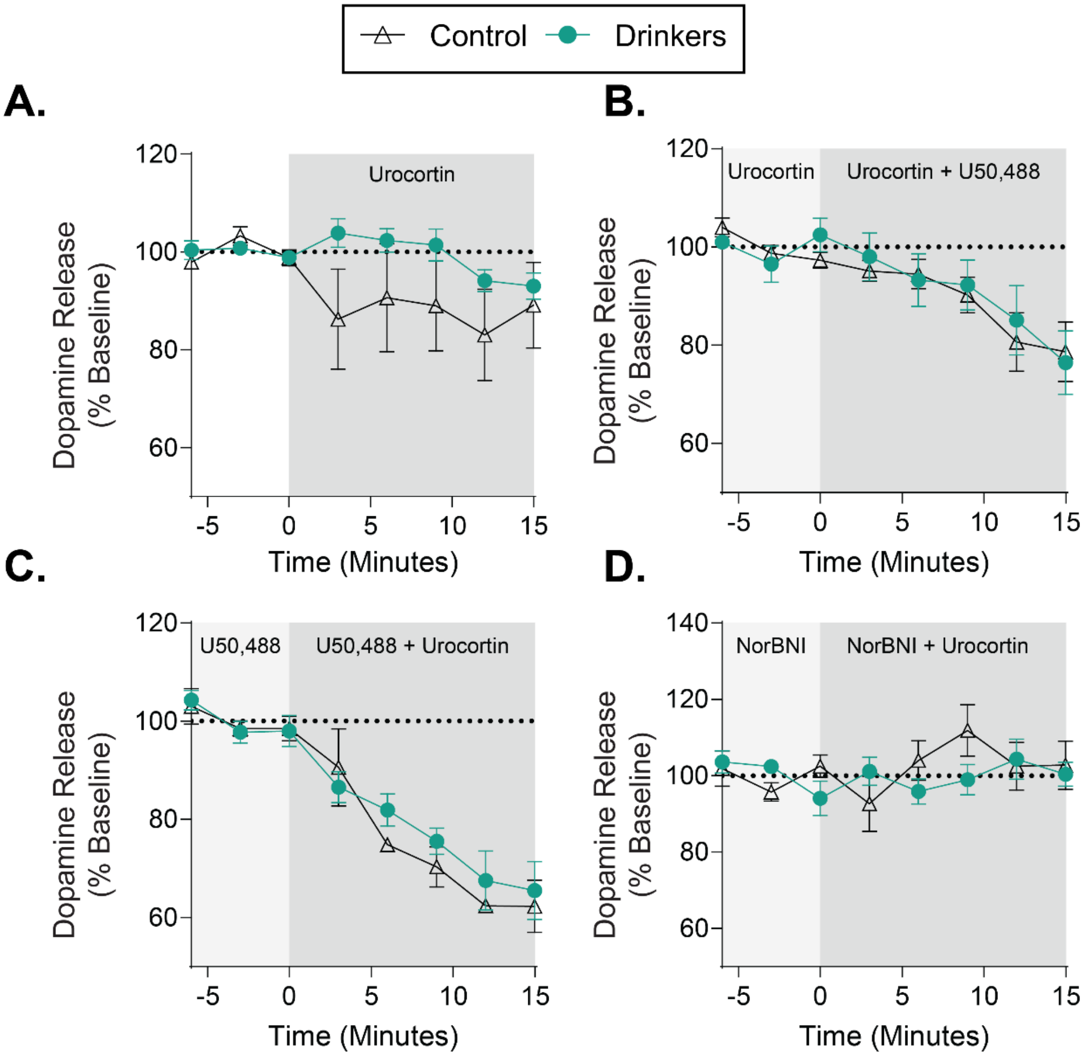

Supplemental Figure 9. Corticotrophin releasing factor (CRF) receptor-mediated inhibition of dopamine release requires kappa opioid receptor co-activation. (A) Urocortin, a CRF receptor agonist, had no effect on accumbal dopamine release in drinkers or controls. (two way ANOVA; time: $F_{1.572, 22.01} = 2.340$, $p = 0.1290$; group: $F_{1, 14} = 1.358$, $p = 0.2633$; time x group: $F_{7, 98} = 1.525$, $p = 0.1678$). **(B)** Consecutive bath application of U50,488, a kappa opioid receptor agonist, decreased dopamine release in both groups similarly, demonstrating a loss of supersensitization of the receptor in the presence of CRF receptor activation (two way ANOVA; time: $F_{2.266, 31.72} = 11.67$, $p < 0.0001$; group: $F_{1, 14} = 0.03315$, $p = 0.8581$; time x group: $F_{7, 98} = 0.4261$, $p = 0.8838$). **(C)** When a kappa opioid receptor agonist was present, the addition of urocortin further decreased dopamine release in drinkers and controls (mixed effects analysis; time: $F_{2.923, 28.39} = 35.53$, $p < 0.0001$; group: $F_{1, 10} = 0.4990$, $p = 0.4961$; time x group: $F_{7, 68} = 0.5462$, $p = 0.7964$)**. (D)** Urocortin had no effect on dopamine release when applied in the presence of a kappa opioid receptor antagonist, norbinaltorphimine (NorBNI) (mixed effects analysis; time: $F_{7, 72} = 0.8132$, $p = 0.5793$; group: $F_{1, 11} = 0.2384$, $p = 0.6349$; time x group: $F_{7, 72} = 1.438$, $p = 0.2037$). Values indicate mean ± SEM. All statistical tests were two-tailed. (controls: n = 8; drinkers: n = 8)
