# Supplementary material for: Synchrony between midbrain gene transcription and dopamine terminal regulation is modulated by chronic alcohol drinking
Source: Nat Commun. 2025 Feb 25;16:1944. doi: 10.1038/s41467-025-56715-y (PMC11850823; doi:10.1038/s41467-025-56715-y)
Supplement: Supplementary file 3 — Reporting Summary [file 41467_2025_56715_MOESM3_ESM.pdf]

nature portfolio

Corresponding author(s): Cody Siciliano

Last updated by author(s): Dec 3, 2024

Reporting Summary

Nature Portfolio wishes to improve the reproducibility of the work that we publish. This form provides structure for consistency and transparency in reporting. For further information on Nature Portfolio policies, see our Editorial Policies and the Editorial Policy Checklist.

Statistics

For all statistical analyses, confirm that the following items are present in the figure legend, table legend, main text, or Methods section.

n/a	Confirmed	
☐	☒	The exact sample size (*n*) for each experimental group/condition, given as a discrete number and unit of measurement
☐	☒	A statement on whether measurements were taken from distinct samples or whether the same sample was measured repeatedly
☐	☒	The statistical test(s) used AND whether they are one- or two-sided<br>*Only common tests should be described solely by name; describe more complex techniques in the Methods section.*
☐	☒	A description of all covariates tested
☐	☒	A description of any assumptions or corrections, such as tests of normality and adjustment for multiple comparisons
☐	☒	A full description of the statistical parameters including central tendency (e.g. means) or other basic estimates (e.g. regression coefficient) AND variation (e.g. standard deviation) or associated estimates of uncertainty (e.g. confidence intervals)
☐	☒	For null hypothesis testing, the test statistic (e.g. *F*, *t*, *r*) with confidence intervals, effect sizes, degrees of freedom and *P* value noted<br>*Give P values as exact values whenever suitable.*
☐	☒	For Bayesian analysis, information on the choice of priors and Markov chain Monte Carlo settings
☐	☒	For hierarchical and complex designs, identification of the appropriate level for tests and full reporting of outcomes
☐	☒	Estimates of effect sizes (e.g. Cohen's *d*, Pearson's *r*), indicating how they were calculated

Our web collection on statistics for biologists contains articles on many of the points above.

Software and code

Policy information about availability of computer code

Data collection	For acquisition of fast-scan cyclic voltammetry data, Demon Voltammetry and Analysis software was used. Behavioral measures of subjects were recorded using custom hardware and programming with a National Instruments interface and Labview software, as previously described.
Data analysis	For analysis of fast-scan cyclic voltammetry data, Demon Voltammetry and Analysis software was used. Statistical analysis of voltammetry and behavioral measures was done in GraphPad Prism (V10). Gene expression analysis was conducted using the Biojupies analysis package, Trimgalore v0.6.7, Salmon v1.9.0, DESeq2 v1.36.0, the WGCNA R package, and the R package cytoHubba. No custom software was used for analysis.

For manuscripts utilizing custom algorithms or software that are central to the research but not yet described in published literature, software must be made available to editors and reviewers. We strongly encourage code deposition in a community repository (e.g. GitHub). See the Nature Portfolio guidelines for submitting code & software for further information.

Data

Policy information about availability of data

All manuscripts must include a data availability statement. This statement should provide the following information, where applicable:

- Accession codes, unique identifiers, or web links for publicly available datasets
- A description of any restrictions on data availability
- For clinical datasets or third party data, please ensure that the statement adheres to our policy

The sequencing data generated in this study have been deposited in the GEO database under accession code GSE244557 [https://www.ncbi.nlm.nih.gov/geo/query/acc.cgi?acc=GSE244557]. The voltammetry data generated in this study are provided in the Source Data file.

Research involving human participants, their data, or biological material

Policy information about studies with human participants or human data. See also policy information about sex, gender (identity/presentation), and sexual orientation and race, ethnicity and racism.

Reporting on sex and gender	N/A
Reporting on race, ethnicity, or other socially relevant groupings	N/A
Population characteristics	N/A
Recruitment	N/A
Ethics oversight	N/A

Note that full information on the approval of the study protocol must also be provided in the manuscript.

Field-specific reporting

Please select the one below that is the best fit for your research. If you are not sure, read the appropriate sections before making your selection.

☒ Life sciences ☐ Behavioural & social sciences ☐ Ecological, evolutionary & environmental sciences

For a reference copy of the document with all sections, see nature.com/documents/nr-reporting-summary-flat.pdf

Life sciences study design

All studies must disclose on these points even when the disclosure is negative.

Sample size	Due to the scarcity of the rhesus macaque model species, a power calculation was not conducted; instead, all subjects available to us were used.
Data exclusions	If evoked dopamine release was below detection thresholds or failed to stabilize (three consecutive collections with <10% variance) that slice was excluded from analysis. These criteria were predetermined.
Replication	Behavioral measures were replicated between the two cohorts included in this study. All replication attempts were successful.
Randomization	Subjects were randomly assigned to one of three groups: drinkers, caloric controls, and housing controls. Housing controls were kept in identical housing conditions to drinkers but no drinking procedure was performed. Each caloric control was yoked to a randomly assigned drinker subject such that they were allowed to self-administer a maltodextrin solution isocaloric to the amount of ethanol consumed by their yoked counterpart.
Blinding	Investigators were blind to group assignment during data collection and analysis of voltammetry data and sequencing. Behavioral experiments were performed and analyzed by separate experimenters blinded to both other outcome measures.

Reporting for specific materials, systems and methods

We require information from authors about some types of materials, experimental systems and methods used in many studies. Here, indicate whether each material, system or method listed is relevant to your study. If you are not sure if a list item applies to your research, read the appropriate section before selecting a response.

Materials & experimental systems

n/a	Involved in the study
☒	☐ Antibodies
☒	☐ Eukaryotic cell lines
☒	☐ Palaeontology and archaeology
☐	☒ Animals and other organisms
☒	☐ Clinical data
☒	☐ Dual use research of concern
☒	☐ Plants

Methods

n/a	Involved in the study
☒	☐ ChIP-seq
☒	☐ Flow cytometry
☒	☐ MRI-based neuroimaging

Animals and other research organisms

Policy information about studies involving animals; ARRIVE guidelines recommended for reporting animal research, and Sex and Gender in Research

Laboratory animals	Subjects were 28 male rhesus macaques (Macaca mulatta) between the ages of 7 and 8.3 years at the time of necropsy.
Wild animals	This study did not involve wild animals.
Reporting on sex	This study design only used males thus findings are only applicable to males.
Field-collected samples	This study did not involve samples from the field.
Ethics oversight	All procedures were conducted in accordance with the Guide for the Care and Use of Laboratory Animals and approved by the Oregon National Primate Research Center Institutional Animal Care and Use Committee.

Note that full information on the approval of the study protocol must also be provided in the manuscript.

Plants

Seed stocks	n/a
Novel plant genotypes	n/a
Authentication	n/a
